# A Case of Scurvy Associated With Intracerebral Hemorrhage in a Patient With Alcohol Use Disorder

**DOI:** 10.7759/cureus.54777

**Published:** 2024-02-23

**Authors:** Yohei Masuda, Kuniyasu Saigusa, Yasuhiro Norisue

**Affiliations:** 1 Department of Emergency and Critical Care Medicine, Tokyo Bay Urayasu Ichikawa Medical Center, Urayasu, JPN; 2 Department of Internal Medicine, Tokyo Bay Urayasu Ichikawa Medical Center, Urayasu, JPN; 3 Department of Neurosurgery, Tokyo Bay Urayasu Ichikawa Medical Center, Urayasu, JPN

**Keywords:** intracerebral hemorrhage, diagnose, alcohol use disorder (aud), vitamin c deficiency, scurvy

## Abstract

Vitamin C deficiency, also known as scurvy, causes abnormalities in connective tissues and varied symptoms. We describe a patient with putaminal hemorrhage, a very rare presentation of scurvy. A 39-year-old man presented with weakness in the left arm and left leg. Right putaminal hemorrhage was initially diagnosed, and he underwent evacuation of the intracerebral hemorrhage. Scurvy was suspected when repeated physical examinations revealed a bleeding tendency and multiple untreated dental caries, missing teeth, and gingivitis. A diagnosis of scurvy was further supported by the patient’s history of smoking, alcohol use disorder, poor diet, and low plasma vitamin C concentration. After receiving oral nutritional supplementation including vitamin C, the bleeding tendency quickly improved. This case highlights the importance of including scurvy in a differential diagnosis for patients with bleeding tendencies, especially those with a poor diet or unknown dietary history. Empirical administration of vitamin C is a reasonable treatment.

## Introduction

Vitamin C deficiency causes abnormalities in connective tissues throughout the body [1], a condition known as scurvy. Although many clinicians consider scurvy to be a disease of the past in high-income countries, persons with a poor diet can develop vitamin C deficiency [2] and scurvy. We report a very rare presentation of scurvy associated with intracerebral hemorrhage in a man with alcohol use disorder.

## Case presentation

A 39-year-old man presented with weakness in the left arm and left leg. We could not obtain a history because of his altered mental status and the absence of witnesses. His colleague called emergency services because the patient was absent without leave and could not be contacted. Paramedics found him on the floor, with no evidence of trauma. The patient is an office worker, and his colleague reported that his past medical history was unclear and that he was a heavy drinker. There was no evidence of illicit drug use.

**Figure 1:**
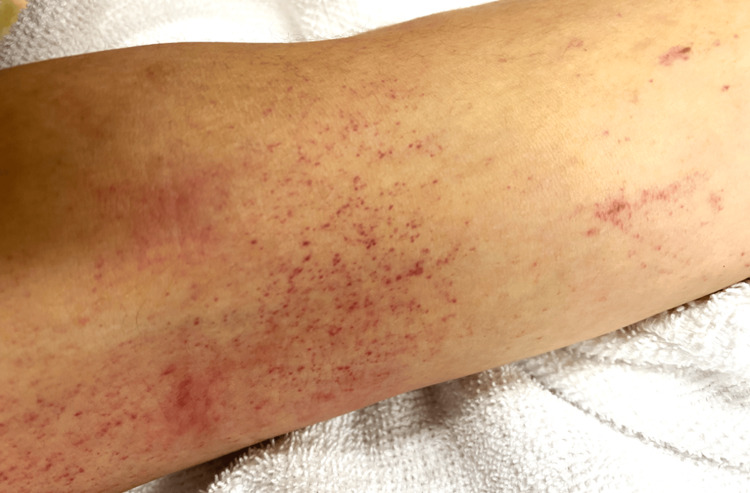
Photograph of petechiae on the left upper arm

On evaluation in the emergency department, the patient’s oral temperature was 37.4°C, the heart rate was 92 beats per minute, the blood pressure was 156/115 mmHg, the respiratory rate was 20 breaths per minute, and the oxygen saturation was 98% while he was breathing ambient air. His body weight was 60 kg and his body mass index was 20.8. He appeared unwell. Examination revealed numerous petechiae over his entire body, with a concentration in the area of the left upper arm typically covered by a blood pressure cuff (Figure 1). A cardiac examination showed no murmurs, and the lungs were clear to auscultation. Bowel sounds were normal. His arms and legs were warm, with no dependent edema. Neurologic examination revealed a Glasgow Coma Scale score of 14 (E4V4M6), weakness of the left arm and left leg, right conjugate eye deviation, and unilateral spatial neglect.

**Figure 2:**
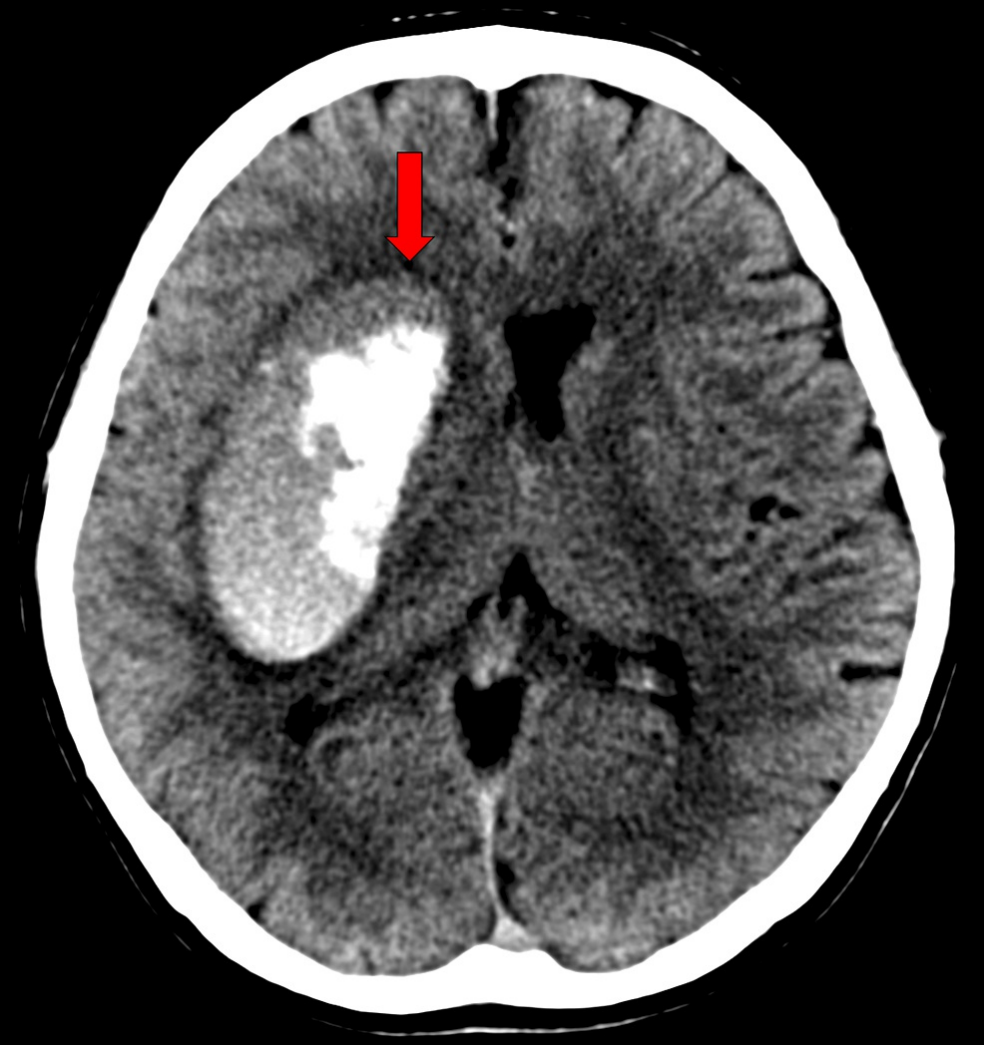
Cranial CT scan showing a right putaminal hemorrhage with compression of the right lateral ventricle and midline shift

The white cell count was 12,000 per milliliter, the hemoglobin level was 17.0 g per deciliter, the mean corpuscular volume was 99.2 fl, and the platelet count was 212,000 per microliter. The results of a basic metabolic panel including blood glucose were within normal limits. The alanine aminotransferase level was 40 IU per liter (normal range, 8 to 42 IU per liter), the aspartate aminotransferase level 58 IU per liter (normal range, 13 to 33 IU per liter), and the γ-glutamyl transpeptidase level 272 mg per deciliter (normal range, 10 to 47 mg per deciliter). Levels of total bilirubin, alkaline phosphatase, and albumin were normal. The international normalized ratio was 0.79 and activated partial thromboplastin time was 30.4 seconds. A cranial computed tomography (CT) scan revealed a right putaminal hemorrhage with compression of the right lateral ventricle and midline shift (Figure 2). Cranial CT angiography (CTA) showed no obvious vascular malformation.

**Figure 3:**
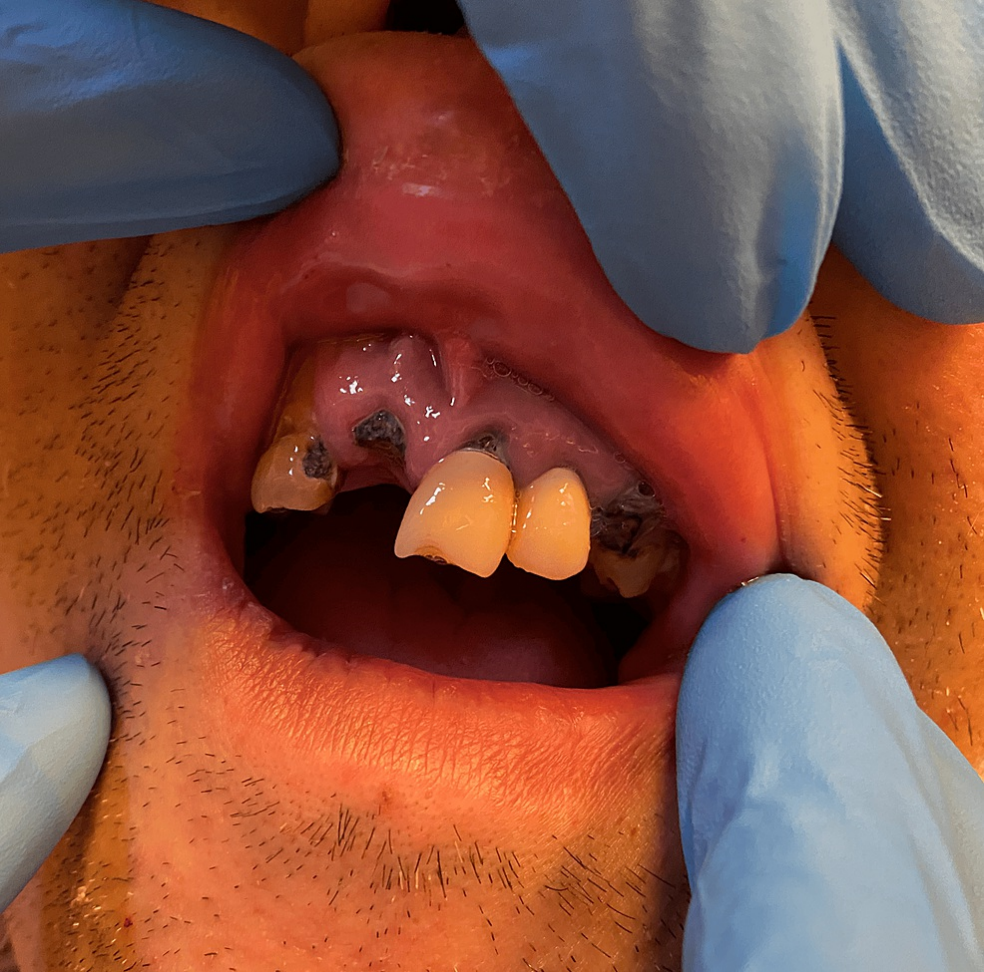
Photograph of oral cavity

Although the patient was disoriented, he was able to understand the need for life-saving surgery and expressed his wish to undergo the procedure. It was therefore determined that he had sufficient decision-making capacity, and oral consent for the surgery was obtained from the patient. He was admitted to the hospital and transferred to the intensive care unit (ICU) before surgery. An intraoral examination before intubation revealed multiple untreated dental caries, missing teeth, and gingivitis (Figure 3). Substantial bleeding from the oral mucosa was observed, even after only slight contact during intubation. After intubation and sedation, the patient underwent successful evacuation of the intracerebral hemorrhage. During surgery, we identified arterial hemostasis but no vascular malformations or lesions. Cauterization was then used to achieve hemostasis.

On hospital day 2, the patient was extubated and began receiving oral nutritional supplementation, including nearly 100 mg/day of vitamin C and increased daily caloric intake; refeeding syndrome was carefully avoided. Petechiae and oral mucosal bleeding were improved on day 8. Because of its absence in the hospital formulary, supplementation with oral vitamin C 200 mg three times a day was delayed. A few days later, plasma vitamin C concentration at admission, 2.1 μg/mL (≈11.9 μmoL/L, normal range >5 μg/mL), was reported by the laboratory. On hospital day 10, his mental status was greatly improved, and he reported a several-year history of alcohol use disorder (estimated alcohol intake >120 g/day) and a 20-pack-year history of smoking. He also reported eating once daily for the past two years, mainly dry snacks such as beef jerky, and almost never eating vegetables or fruit. He had no family history of atherosclerotic cardiovascular disease. In addition to vitamin C supplementation, we advised smoking cessation, abstinence from alcohol, and nutritional guidance as secondary prevention. Additionally, we consulted with a dentist, who assessed the patient's oral hygiene. While hospitalized, the patient continued to have hypertension (blood pressure >140/90 mmHg). Therefore, we initiated oral administration of amlodipine 5 mg before discharge.

Although incomplete left-side paralysis and higher cerebral dysfunction remained, the patient substantially recovered and was transferred to a rehabilitation hospital three weeks after admission.

## Discussion

Scurvy probably contributed to the intracranial hemorrhage in the present patient. Putaminal hemorrhage was initially diagnosed but repeated physical examinations in the ICU revealed a bleeding tendency, without thrombocytopenia or coagulopathy, and oral findings consistent with vitamin C deficiency. These findings, in addition to the presence of alcohol use disorder, strongly suggested scurvy, despite the lack of a dietary history and persons familiar with the patient’s diet. Scurvy was ultimately diagnosed when his dietary history was confirmed and his clinical symptoms improved after vitamin C supplementation.

Scurvy is a clinical diagnosis based on dietary history, clinical manifestations, and rapid improvement with vitamin C supplementation [3]. Clinicians should be aware of risk factors for scurvy, such as alcoholism, low socioeconomic status, and severe psychiatric disease [2]. Clinical manifestations of scurvy begin when fruit and vegetable consumption is severely limited for eight to 12 weeks [2,4]. The early stage of the illness, sometimes referred to as latent scurvy, is characterized by fatigue, dull aches, and weight loss. Latent scurvy may be underreported because it is nonspecific [5]. Prolonged vitamin C deficiency results in scurvy manifestations such as petechiae, gingival hypertrophy and bleeding, gingivitis, and corkscrew hair [6].

Scurvy is an ancient disease that is difficult to diagnose from laboratory findings alone. Clinicians should include scurvy in the differential diagnosis based on patient characteristics such as alcoholism and suspected abnormalities of connective tissue, including bleeding tendency, and obtain a dietary history. A minimum vitamin C dose of 100 mg three times a day is necessary to address the deficiency and replete body stores rapidly. Subjective symptoms improve within one day, petechiae resolve quickly, and most symptoms disappear within a few weeks [7].

Scurvy associated with intracranial hemorrhage is extremely rare. Indeed, only a few case studies during the previous 40 years have reported intracranial hemorrhage due to scurvy [8-10]. 

Hypertensive cerebral hemorrhage is a common cause of putaminal hemorrhage; however, our patient presented with hypertensive intracerebral hemorrhage. Hypertensive cerebral hemorrhage commonly occurs in adults aged approximately 60 years with a long history of hypertension [11], although alcohol use disorder was reported to be a risk factor for hypertensive cerebral hemorrhage in young adults. Although cerebrovascular malformation, such as arteriovenous malformation or cavernous angioma, is a common cause of intracerebral hemorrhage in younger adults [12], CTA and intraoperative findings showed no evidence of cerebrovascular malformation in the present patient. Therefore, we considered factors in addition to hypertension that could have contributed to putaminal hemorrhage. Because of its association with connective tissue defects, scurvy may increase the risk of intracerebral hemorrhage [1]. In addition, a previous cohort study reported a strong inverse correlation between serum vitamin C concentration and the incidence of hemorrhagic intracerebral hemorrhage [13].

Vitamin C deficiency is common among ICU patients with alcohol use disorder [14], but reports of scurvy in the ICU are rare, which highlights the importance of obtaining a dietary history. Clinicians might hesitate to include scurvy in a differential diagnosis without a proper history, and symptoms of scurvy tend to improve when vitamin C deficiency incidentally resolves after receipt of enteral nutrition or food during hospitalization.

## Conclusions

The present case highlights the importance of including scurvy in a differential diagnosis for patients with a bleeding tendency in the absence of thrombocytopenia or coagulopathy, especially for those with a poor diet or unknown dietary history. In such cases, empirical administration of vitamin C is indicated, because of its low cost, safety, and potential for immediate benefit. Notably, this case highlights a potential link between scurvy and intracranial hemorrhage, although hypertension may have been an additional aggravating factor in our patient.
